# Brown algal morphogenesis: atomic force microscopy as a tool to study the role of mechanical forces

**DOI:** 10.3389/fpls.2014.00471

**Published:** 2014-09-17

**Authors:** Benoit Tesson, Bénédicte Charrier

**Affiliations:** ^1^Marine Biology Research Division, Scripps Institution of Oceanography, University of California San DiegoLa Jolla, CA, USA; ^2^Centre National de la Recherche Scientifique-Unités Mixtes de Recherche 8227 Integrative Biology of Marine Models, Station Biologique de RoscoffRoscoff, France; ^3^Sorbonne Universités, Université Pierre-et-Marie-Curie, Unités Mixtes de Recherche 8227 Integrative Biology of Marine ModelsRoscoff, France

**Keywords:** brown algae, *Ectocarpus siliculosus*, morphogenesis, cell wall, mechanical forces, AFM

## Abstract

Over the last few years, a growing interest has been directed toward the use of macroalgae as a source of energy, food and molecules for the cosmetic and pharmaceutical industries. Besides this, macroalgal development remains poorly understood compared to other multicellular organisms. Brown algae (Phaeophyceae) form a monophyletic lineage of usually large multicellular algae which evolved independently from land plants. In their environment, they are subjected to strong mechanical forces (current, waves, and tide), in response to which they modify rapidly and reversibly their morphology. Because of their specific cellular features (cell wall composition, cytoskeleton organization), deciphering how they cope with these forces might help discover new control mechanisms of cell wall softening and cellulose synthesis. Despite the current scarcity in knowledge on brown algal cell wall dynamics and protein composition, we will illustrate, in the light of methods adapted to *Ectocarpus siliculosus*, to what extent atomic force microscopy can contribute to advance this field of investigation.

In addition to cell differentiation, the morphogenesis of multicellular organisms depends both on growth rates and growth directions. As there are now several evidences indicating that mechanical forces are important factors of cell growth and polarity (Braam, 2005; Hamant, 2013), researchers recently started to combine genetic information with biophysical measurements.

In plant organisms, two kinds of mechanical stimuli may influence growth rates and directions. Environmental stimuli like wind, current or rock, were known for a long time to influence plant growth. More recently, several evidences suggested that internal mechanical forces as well, generated by the growth itself, could be considered as morphogenetic factors, which control the spatial organization of the organism like biochemical gradients do (Monshausen and Gilroy, 2009; Hamant and Traas, 2010).

How external or internal mechanical forces impact on cell growth in land plants will likely be covered in this special issue by colleagues. We will present herein how external mechanical factors also contribute to morphogenetic changes in brown algae and we will introduce the method of investigation that we are currently deploying to identify the biophysical and molecular factors at play in this process.

## Impact of mechanical forces in brown algal morphogenesis: themes and variations compared to land plants

Brown algae form a monophyletic group of multicellular organisms, which is phylogenetically divergent from the other groups of macroalgae (red and green) and other multicellular organisms like land plants and metazoans. They also differ from land plants in many ways, and particularly by their habitat and morphologies. They are dominant in the highly variable coastal environment and display very diverse body architectures, from microscopic species to giant kelp of up to 50 m long. Brown algae and land plants live in different environments, each presenting distinct physical constraints. In particular, gravity is less perceived in seawater which is denser than air and provides support to organisms allowing more flexible bodies.

### Impact of external forces

In the ocean, mechanical forces such as currents and tides control both shape and size of macroalgae. Their flexible bodies are particularly suitable to adapt to such changing environments. In particular, macroalgae are capable to rapidly change their morphologies in a reversible way in response to environmental changes. This is actually a key factor for their survival and propagation in the ocean (Koehl, 1984; Dudgeon and Johnson, 1992; Blanchette, 1997). This ability might be attributable to their low body complexity with only a few cells types, in addition to a high responsiveness to external stimuli (Charrier et al., 2012). Adaptation is achieved by a phenomenon called flexible reconfiguration which allows the organisms to reduce drag. Flexible reconfiguration involves shape change and size reduction (Boller and Carrington, 2006; Martone et al., 2012).

On rocky shores water velocity induced by waves ranges from 2 to 25 m.s^−1^ with acceleration exceeding 400 m.s^−2^ (Friedland and Denny, 1995). These speeds could seem rather low compared to the wind but it is without accounting for the density which is about 1000 times higher for water compared to air. Force resulting from a 2 m.s^−1^ current would correspond to a 208 km.h^−1^ wind (Denny and Gaylord, 2002). These particularly turbulent conditions require macroalgae to be flexible and to adapt by changing their morphology if they are to survive. An intrinsic adaptation of algae to mechanical forces is their simple body architecture allowing a very large diversity of shapes and their flexible body. This flexibility is due to material constituting algal cell wall which is characterized by a low stiffness and a high extensibility. Stiffness of algal material is in the range of 1–100 MPa, i.e., about 1000 times softer compared to land plants (Denny and Gaylord, 2002). Adaptation of macroalgae to wave swept environments usually involves narrow, thick, and flat blades while the counterpart in sheltered habitats display wide, thin, and undulated blades. Undulation has been shown to result from differential growth in the center and at the periphery of the blades. Higher longitudinal growth at the periphery than in the center of the blade produces ruffles on the edges due to elastic buckling (Koehl et al., 2008). Undulated, wide blades found in low flow regime increase drag and light interception while the inverse is observed for flat and narrow blades from wave exposed sites (Koehl et al., 2008). Buoyancy is also affected by mechanical stress. Within the same species, and in protected areas, algae develop a specific cell type called “pneumatocysts” increasing buoyancy, while in exposed areas, pneumatocysts are either very small or absent (Stewart, 2006). These morphological variations are due to the plasticity of macroalgae and not only to genetic traits, as algae are able to rapidly change their morphology in response to flow regime changes (Fowler-Walker et al., 2006).

### Potential internal mechanical forces involved in cell growth and cellular responses

Tip-growing brown algae, such as *Sphacelaria* (Order Sphacelariales) and *Ectocarpus* (Ectocarpales) are particularly suitable models to study the role of mechanical forces on cell growth rate and direction. As in the other tip-growing cells, the cylindrical shape of the apical cells results in a stress in the circumferential direction twice as large as in the axial direction (Castle, 1937). Cell wall of tip-growing cells of *Sphacelaria rigidula* consists of four layers: (i) an external thin amorphous layer, (ii) a layer consisting of fibrillar materials embedded in an amorphous matrix, (iii) a layer made of transversally oriented cellulose fibers and (iv) a layer of longitudinally oriented cellulose fibers (Karyophyllis et al., 2000). The external amorphous layer likely consists of amorphous alginates mainly, while the more internal layers are enriched in cellulose. Interestingly, the cell wall was found to be thinner at the tip, consisting only of the first two layers, likely making the wall softer in this region. Moreover, transversal orientation of cellulose fibers at the flank provides resistance to deformation in this direction. To promote tip growth, cells need to modulate the mechanical properties of their cell wall. Two mechanisms are possible: 1- softening of the cell wall at the tip and/or 2- anisotropic organization of the cellulose fibers to resist transversal deformation and favor tip elongation (Mirabet et al., 2011).

#### Softening of the cell wall at the tip

In pollen tube, tip-growth is mediated by secretion of methyl-esterified pectin at the tip and gradual stiffening is achieved by de-esterification of pectin and calcium mediated cross-linking of the carboxyl groups (Rounds and Bezanilla, 2013). No pectin was reported to be present in brown algal cell walls. However, other compounds could fulfill the same role. Brown algal cell wall is composed principally of alginates, sulfated fucans and of a relatively low amount of cellulose (Kloareg and Quatrano, 1988). Alginates are polymers of mannuronic and guluronic acids in various amounts. Interestingly, their properties depends on the relative amount of manuronnans and guluronans, as stiffness increases with increasing guluronan content, the latter forming binding sites for calcium ions, thereby inducing gelation (Draget and Taylor, 2011). Secretion of alginates composed of manuronans at the tip and subsequent epimerization of mannuronans into guluronans by the mannuronan C5 epimerase (MC5E) (Michel et al., 2010) would lead to a softer wall at the tip compared to shank. In addition, recent chemical analyses of brown algal cell walls (Order Fucales) showed that alginates were linked to most phenolic compounds present in the cell wall (Deniaud-Bouët et al., 2014). The progressive linkage of apical newly-deposited alginates to phenolic compounds mediated by the activity of extracellular haloperoxidases could increase stiffness in the flanks of the cell during tip-growth. Noteworthily, fucose-containing sulfated polysaccharides (FCSP) were also shown to be tightly linked to cellulose and cell wall proteins, but these would be more involved in the regulation of water retention at the cell surface than to cell wall mechanical resistance (Deniaud-Bouët et al., 2014).

#### Actin-mediated cellulose orientation

At the cellular level, localization of actin microfilaments (MF) and microtubules (MT) as well as the use of cytoskeleton inhibitors on the brown algae *Sphacelaria rigidula* revealed major differences with land plants and some similarities with diatoms, which are the closest relative to brown algae (Katsaros et al., 2006). Indeed, like in diatoms and animal cells, cytokinesis requires the formation of an actin plate, and, in contrast with land plants, cellulose microfibril deposition seems to be under the control of actin MF and not of MT (Katsaros et al., 2006; De Martino et al., 2009). Localization of actin MFs in *Sphacelaria* apical cells showed that they are oriented in the longitudinal direction except at the base of the apex, where actin is organized as a ring in the transverse section, and at the tip of the apex itself, where MFs are randomly oriented. Orientation of MFs corresponds to the orientation of the cellulose fibers in the inner layer of the cell wall. Furthermore, treatment with cytochalasin D, an inhibitor of actin polymerization induced tip growth arrest and altered orientation of newly deposited cellulose fibrils (Karyophyllis et al., 2000; Katsaros et al., 2003).

Altogether, both local biochemical modifications of amorphous cell wall materials and actin-mediated cellulose microfibril orientation could control cell wall stiffness and promote the anisotropic growth of tip-growing cells in brown algae.

## Measuring mechanical forces experienced by brown algal cells

### Available technical tools and application to plant cells

Several tools allowing the measurement of cell mechanical properties have been developed including single cell compression, ball tonometry, microindentation, Cellular Force Microscopy (CFM), Nanoscale Dynamic Mechanical Analysis (NanoDMA), and Atomic Force Microscopy (AFM). One of the differences between these tools is the size of the probe which is an important parameter for the study of microorganisms. Ball sizes in the ball tonometry method range from 300 to 5000 μm and microindentation and CFM probes are 2–11 μm. NanoDMA and AFM have a wider range of probes from 10 nm (pyramidal tip) to several microns (spherical probes). For more advanced reviews see Milani et al. (2013) and Routier-Kierzkowska and Smith (2013).

Recently, properties of land plant cell walls were monitored using AFM (Milani et al., 2011; Fernandes et al., 2012) and changes in mechanical properties were correlated with growth and activity of cell wall enzymes (Milani et al., 2011; Peaucelle et al., 2011).

Cell wall is not homogeneous. Depending on the size of the probes, of the indentation depth, and of the turgor pressure, the contribution of underlying materials might influence the measured stiffness. On the one hand, by dividing force indention curves into segments, Roduit et al. (2009) developed “AFM tomography” and used it to differentiate layers with distinct mechanical properties within the cell wall of *Arabidopsis* cell suspension (Radotić et al., 2012).

On the other hand, nanoindentation and CFM combined with finite element modeling were recently used to measure turgor pressure in plant cells, and allowed to complement data obtained using pressure probes or the plasmolysis limit method. Indentations were realized at various depths and in different conditions of turgor pressure to isolate cell wall properties *per se* from turgor pressure (Forouzesh et al., 2013; Vogler et al., 2013).

Simple indentation shows the instantaneous elastic behavior. However, the cell wall is a more complex material with viscoelastic properties, meaning that deformation is time dependent. Elastic behavior is associated with the stretching of bonds while viscosity is associated with the diffusion of molecules in amorphous polymers. Various methods can be used to determine viscoelastic properties including Dynamic Mechanical Analysis (DMA), stress relaxation, and creep. Determination of viscoelastic properties of plant cell walls revealed distinct properties in mutants, ecotypes, and at different maturity stages (Hayot et al., 2012; Forouzesh et al., 2013).

### AFM to study mechanical forces in the brown alga ectocarpus

In order to start studying the role of physical forces in brown algal cell growth and morphogenesis, we are currently investigating the mechanical properties of the tip-growing filamentous alga *Ectocarpus siliculosus*. Given the size of *Ectocarpus* cells, nanoindentation tools such as AFM are better suited.

#### AFM principle and applications

**Figure 1:**
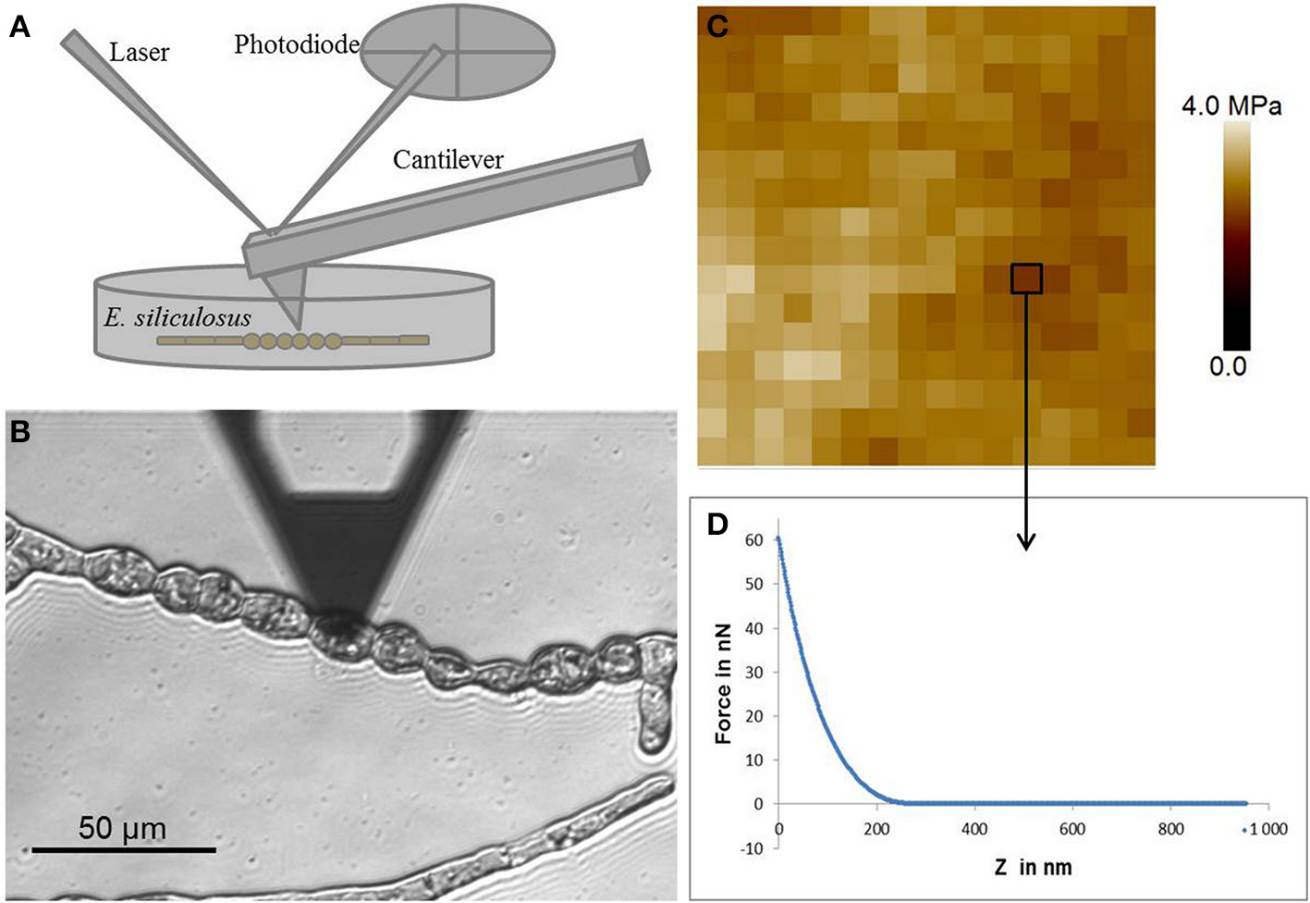
**Atomic force microscopy. (A)** Schematic representation of the AFM setting. **(B)** Optical micrograph of the AFM cantilever located at the surface of a round cell of *E. siliculosus* filament. **(C)** Example of force curve, x axis represents the z motion of the cantilever and y axis represents the force applied at the surface of the cell. **(D)** Elasticity map of a 1 μm^2^ area consisting of an array of 16 by 16 force curves.

AFM is a surface scanning technique. Originally it was mainly used to image the surface of samples but it evolved into a versatile tool with a wide range of applications (Muller and Dufrene, 2011). Principle is based on the scanning of a surface with a probe consisting in a tip located at the end of a cantilever (Figure 1A). Compared to the other techniques, AFM displays the advantage of allowing surface imaging with a very high resolution in native (hydrated) conditions. Tip sizes can vary from a few nanometers to few tens of nanometers and bead sizes from 100 nm to 20 μm. The coupling to an inverted microscope allows highly resolutive localization and observation of the sample prior to analysis with the AFM, which is a prerequisite for the study of microorganisms. In order to measure cell mechanical properties, force deformation curves are acquired by pushing on the cell surface. From this curve, elastic modulus (E) is determined by fitting the curve using mechanical models for elastic materials. The most well-known is the Hertz model which was developed for spherical indenters implying that the deformation depth is much smaller than the indenter radius (Hertz, 1882). The Hertz model was modified by Sneddon to be adapted to conical indenters implying that the indentation depth is greater than the tip radius of the indenter (Sneddon, 1965). Viscoelastic properties can be determined using AFM by performing relaxation or creep tests. Resulting curves are then fitted with a model consisting in various arrangements of springs and dash pots representing elastic and viscous behavior respectively.

Two main limitations are inherent to the use of AFM: (i) the sample should adhere to a substrate and (ii) be accessible to the tip. Hence, in plants, AFM was particularly adapted to study the cell wall of the moss *Physcomitrella* filaments (Wyatt et al., 2008), the angiosperm pollen tubes (Wu et al., 2008) and hypocotyls (Marga et al., 2005). In addition, as AFM can be carried out in liquid medium, it is particularly suitable for algae, and was used in diatoms, both at the surface of live cells (Francius et al., 2008) and from extracted cell walls (Tesson and Hildebrand, 2010a,b, 2013).

#### Application of AFM to ectocarpus filaments

**Figure 2:**
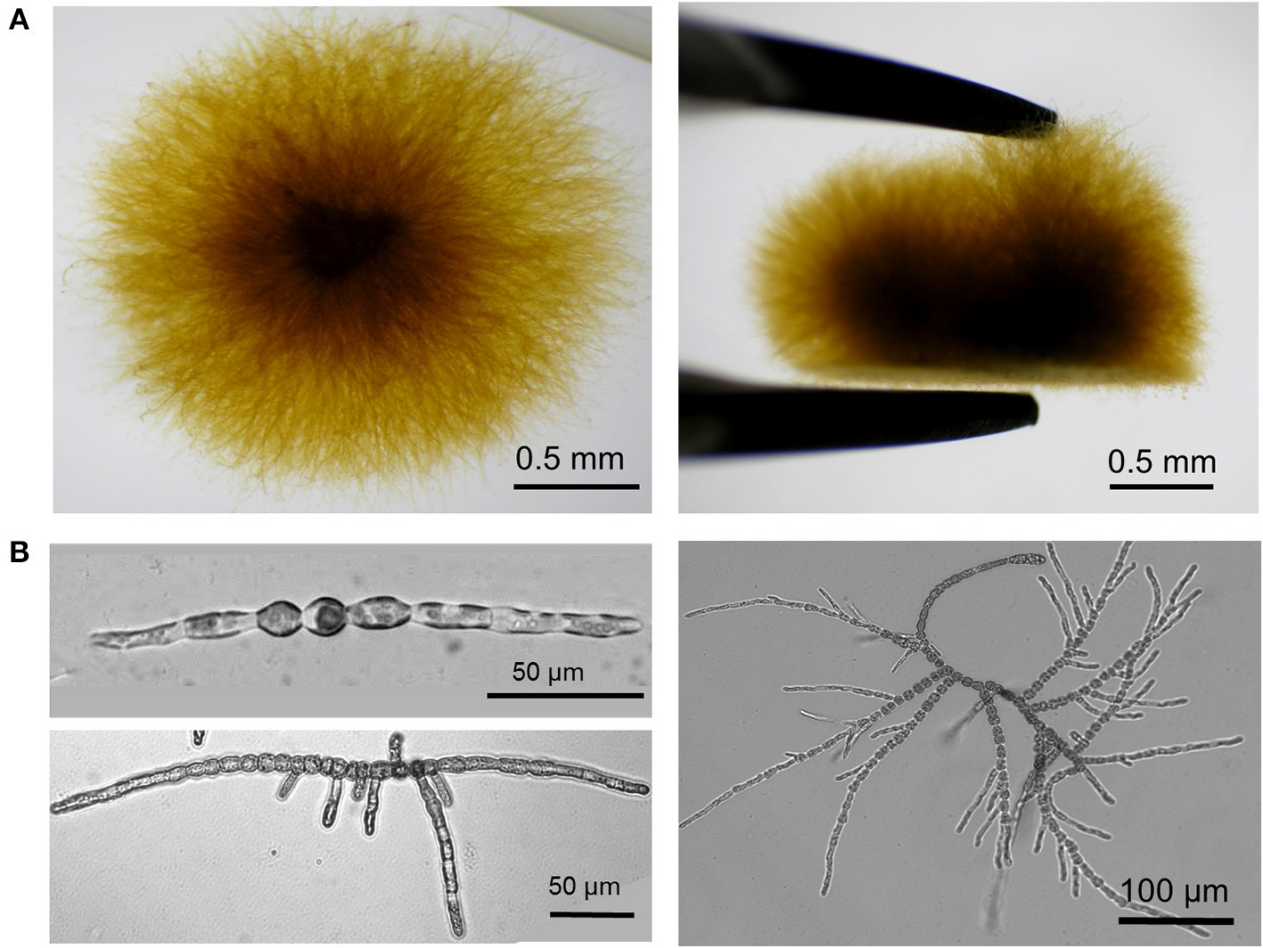
**Developmental pattern of *E. siliculosus*. (A)** Top (left) and side (right) views of an *Ectocarpus* sporophyte grown in laboratory condition for 1 month in sea water. For the side view, the algal body was taken off from the bottom of the Petri dish with pliers. **(B)** *Ectocarpus* sporophyte is formed of branched uniseriate filaments. In the early stages, the sporophyte differentiates only two cell types: round cells localized in the center of the filament and elongated cells at the apices. Initially, mito-spores attach to the substrate and divide by successive uniaxial mitoses to form the young sporophyte filament (top left). Young sporophytes grow apically by both elongation and division of the apical elongated cells, which eventually differentiate into round cells. Branching takes place by emergence of secondary filaments preferentially on round cells in the center of the filament (Le Bail et al., 2008) (bottom left). Re-iteration of this process on each newly formed filament gives rise to a highly branched organism (bottom right and Panel **A**).

*Ectocarpus* is a filamentous brown algae growing attached to a substrate (a Petri dish in laboratory conditions) which makes cells easily accessible to experimentation. However, at late stages (2–3 weeks after spore germination), secondary filaments grow in the “z” direction, which makes the primary filaments less accessible (Figure 2A). For this reason AFM analysis in *Ectocarpus* was so far only carried out at early stages when the body was two-dimensional (Figures 1B, 2B). *E. siliculosus* differentiates two cell types, which are differentially located along the filament (Le Bail et al., 2008), we used an AFM coupled to an inverted microscope (Bioscope catalyst, Bruker) to precisely localize the AFM tip over the filament.

In details, *E. siliculosus* samples were prepared from spore release in small Petri dishes (60 mm diameter, fitting on the microscope stage) in the presence of 10 mL sea water (for detailed description, see Le Bail and Charrier, 2013). Filaments adhered and grew to the bottom of the Petri dish until the stage of interest was reached. Force indentation curves were acquired using ScanAsyst fluid cantilevers (Bruker) with a spring constant of approximately 1.5 N/m. Cantilever was calibrated by measuring deflexion sensitivity and spring constant. The deflexion sensitivity was determined by recording a force curve on a hard surface in seawater. The spring constant was then measured using the thermal tune method in air, which consists in the determination of the resonance frequency of the cantilever. A maximum load of 60 nN was used leading to deformations between 100 and 300 nm (depending on cells) which was below cell wall thickness (approximately 400 nm) (Figure 1C). Cartographies of elasticity were obtained by fitting the curves with the Sneddon model (Figure 1D). So far, measurements on several filaments from different Petri dishes allowed to obtain reproducible and consistent data on different *Ectocarpus* cell types (Tesson and Charrier, in prep), showing the suitability of this technique to this alga. Further experiments will be necessary to obtain a full description of the mechanical landscape in *Ectocarpus* filament.

## Perspectives

**Figure 3:**
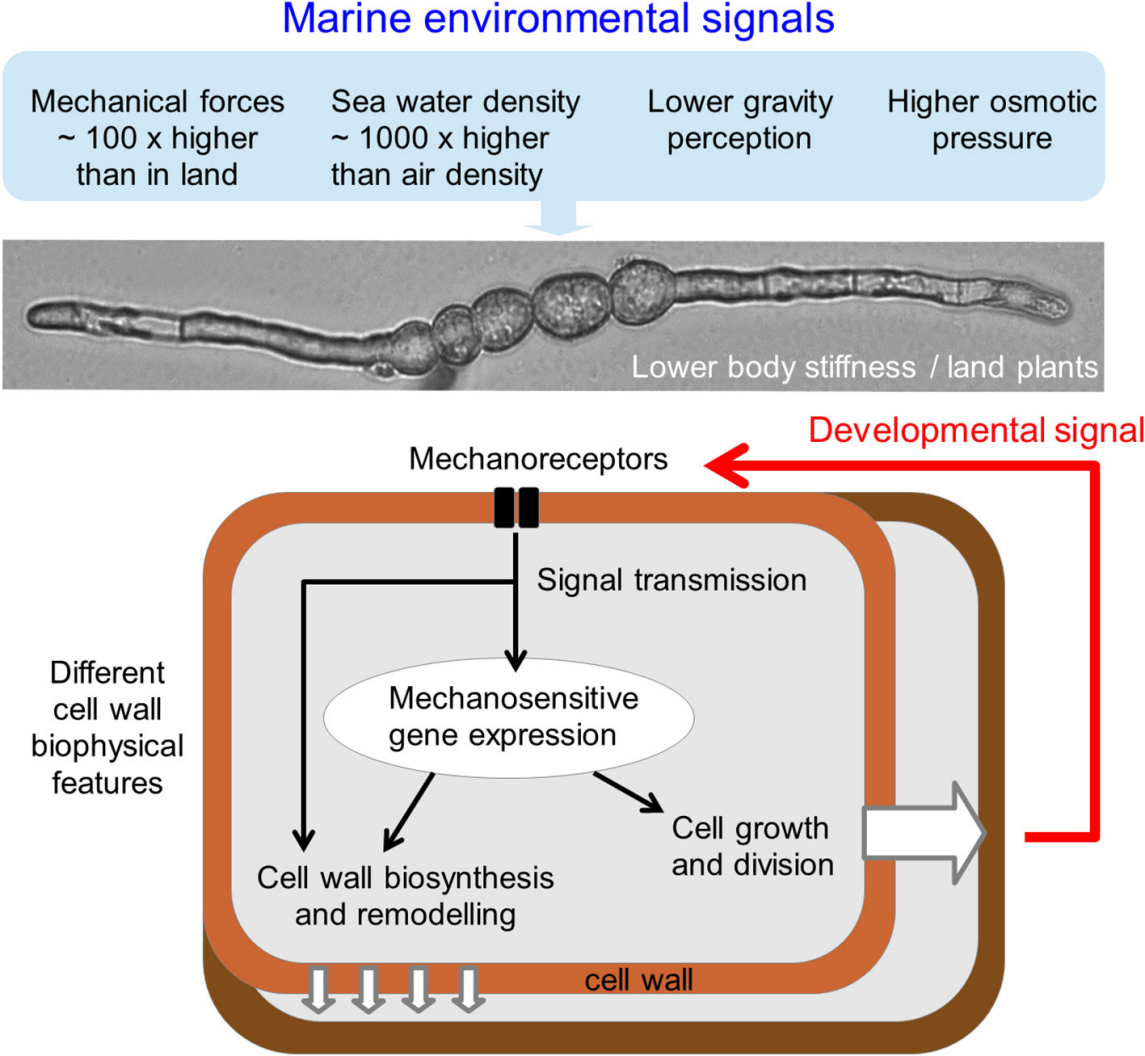
**Schematic representation of cell responses to mechanical signals**. The specific environmental and cellular features of marine brown algae likely make molecular responses to mechanical signals different from land plants. In addition to the higher external osmotic pressures, mechanical forces and density and to the lower gravity perception, brown algal cell wall composition, structure, and physical properties differ from land plants. Nevertheless, similarly to land plants, primary molecular factors involved in perception and response to mechanical signals likely include transmembrane mechanoreceptors and signaling pathway factors. Resulting activation of the nuclear (mitosis and gene expression) and cell wall (biosynthesis and remodeling) machineries will allow relevant physiological and mechanical adjustment to the external mechanical constraints. Modification of the spatial and biophysical extracellular conditions due to the growth within the algal thallus will also likely generate mechanical signals perceived by similar or distinct mechanosensors.

Characterization of the mechanical properties of cells along the filament will allow to determine the role of mechanical constraints in cell growth and differentiation. This information will be then correlated with biochemical and genetic data. Beside the description of the physical properties of the brown algal cell wall using AFM as a technical tool, the molecular factors involved in the mechanosensitive pathways should be investigated (Figure 3). By looking at sequence similarities between the genome of *E. siliculosus* (Cock et al., 2010) with land plants, bacteria, and animal proteins, putative genes involved in mechanosensing were identified (Charrier et al., 2012). These candidate genes include transmembrane receptor-like kinase (RLK), mechanosensitive channels (MscS), and transmembrane proteins showing similarities with alpha integrins. Alpha integrins are usually found only in metazoans. Additionally, in a study of auxin signaling in *E. siliculosus*, the gene *EsGRP1* was shown to be over-expressed in the presence of exogenous auxin and differentially expressed in morphogenetic mutants. Interestingly, this protein contains extensin-like domains with RGD motifs and glycine-rich regions (Le Bail et al., 2010). Extensins are plant cell wall proteins involved in cell wall formation (Lamport et al., 2011), and a glycine-rich protein (AtGRP3) was shown to bind and regulate the wall associated kinase WAK1 (Park et al., 2001). RGD motifs are known to be involved in interaction with integrins (Takada et al., 2007), and despite the absence of true integrin homologs, the RGD peptide was shown to interfere with several developmental events in plants (Jaffe et al., 2002).

However, these are only candidates based on sequence similarities, and mechanosensors specific for brown algae will be identified more likely by functional studies. A bank of morphogenetic mutants grown in standard conditions was generated in *Ectocarpus* (Le Bail and Charrier, 2013). Several mutants, for which altered physical properties of the cell wall could easily account for their phenotypes, include those displaying irregular cell contours or cell bursting. The identification of the mutated genes responsible for these phenotypes together with the detailed study of the cell wall structure and of its mechanical properties by AFM will allow to establish for the first time a functional link between genes and the overall composition and organization of the cell wall in brown algae and its function in cell shape. In parallel to studying morphological mutants growing in standard conditions, mutants subjected to mechanical stress should be investigated. Morphological responses of *Ectocarpus* to artificial sea currents mimicked by rotational shear stresses generated by an orbital shaker (Yun et al., 2002; Dardik et al., 2005; Sumpio et al., 2005) should be screened in order to select for mechanically hypo- or hyper-sensitive mutants. Whether these mechanosensors share similarities with land plant or even metazoan counterparts, or are specific to brown algae, will then be determined.

### Conflict of interest statement

The authors declare that the research was conducted in the absence of any commercial or financial relationships that could be construed as a potential conflict of interest.

## Abbreviations

AFM: Atomic Force Microscopy
MF: Microfilament
MT: Microtubule.
